# 

**Published:** 1889-05

**Authors:** 


AN INTERNATIONAL CONGRESS OF THERAPEUTICS AND MATERIA MEDICA will hold sessions from the 1st to the 5th of August, 1889, at the Hôtel des Sociètiès Savantes, No. 28 Rue Serpente, Paris, in connection with the Exposition. The Committee of Organization is composed of MM. Moutard-Martin, President; Dujardin-Beaumetz, Vice-President; Constantine Paul, Secretary-General; P. G. Bardet, Adjunct Secretary-General; Labbè, Secretary of the Section of Therapeutics; and Blondel, Secretary of the Section of Materia Medica. Physicians, pharmacists, and veterinarians, upon payment of ten francs are admitted to membership. Titles of papers should be sent to Dr. Bardet before May 15th, at 119 bis, Rue Notre-Dame-des-Champs, and to whom all communications relating to the Congress should be addressed.

---

THE BUFFALO MEDICAL AND SURGICAL ASSOCIATION will hold its regular monthly meeting at No. 9 West Mohawk street, Tuesday, May 7, 1889, at 8.30 o'clock P. M.

At the annual meeting April 2, 1889, the following named were elected officers for the ensuing year: President, A. A. Hubbell; Vice President, M. B. Folwell; Secretary, W. H. Bergtold; Treasurer, F. E. L. Brecht; Librarian, Lucien Howe.

Programme, May 7th: Paper on "Gynecology," by Dr. M. Hartwig; inaugural address by the President, subject: "The Buffalo Medical and Surgical Association—Retrospective and Prospective." The two only living representatives of the founders of the Association will be asked to sit with the President on this occasion.

---

A CIRCULAR has been issued announcing an International Congress of Otology and Laryngology, to be held in Paris from the 16th to the 21st of September, 1889. The admission fee has been fixed at twenty francs. The hope is expressed that the Congress will be attended by many interested in these departments of medicine, and that the meeting will be one of scientific importance. All communications relating to this meeting should be addressed to the Secretary of the Committee of Organization, M. le docteur Loewenberg, rue Auber, 15 à Paris.

---

THE AMERICAN SOCIETY OF MICROSCOPISTS will hold its next annual meeting in this city, August 20, 21, 22, and 23, 1889. It is expected that this will be an unusually well-attended and interesting meeting. The local society and all physicians should unite in contributing to the comfort of guests and general success of this distinguished society.

---

THE PENNSYLVANIA STATE MEDICAL SOCIETY will hold its annual meeting at Pittsburgh, June 4, 5, 6, and 7, 1889.

THE ONTARIO MEDICAL ASSOCIATION will hold its next annual meeting in Toronto, June 5 and 6, 1889. Dr. Roswell Park, of this city, will read a paper on "The Radical Cure of Hernia."

---

THE MICHIAGN STATE MEDICAL SOCIETY will hold its twenty-fourth annual meeting in Kalamazoo, May 9 and 10, 1889.

---

THE STATE MEDICAL SOCIETY OF NEBRASKA will hold its annual meeting at Kearney, commencing May 21, 1889.

---

THE OHIO STATE MEDICAL SOCIETY will hold its annual meeting at Youngstown, beginning May 22, 1889.

### Literary and Other Notes.

Medical and Surgical Register of the United States.—The second edition will contain a list of the physicians and surgeons in the United States, arranged alphabetically; also arranged by States and cities or towns, showing method of practice, date and college of graduation, post-office address, with population and location.

Also lists of existing and extinct medical colleges in the United States and Canada, with location, officers, number of professors, lecturers, demonstrators, etc., the various medical societies, hospitals, sanitariums, asylums, and other medical institutions, Boards of Health, a synopsis of the laws of registration, and other laws relating to the profession in each State, medical journals, with names of editors, frequency of publication, and subscription rates, official list of officers of the Medical Departments U. S. Army, Navy, Marine and Hospital service, roster of examining surgeons of the U. S. Pension department, a descriptive sketch of each State and territory, embodying such matters as location, boundaries, extent in miles and acres, latitude and longitude, statistics relating to climate, temperature, rate of mortality, number of deaths from consumption, etc., the names and locations of all the best known mineral springs, full particulars of all national associations and societies relating to medicine and surgery. Complete in one volume, subscription price, $5.00; to non-subscribers, $7.00. To insure correct insertion of your name, it is essential that you give the information to our agent yourself. R. L. Polk & Co., Publishers, Philadelphia, Pa.

---

ARCHIVES OF PEDIATRICS.—The April number of the *Archives of Pediatrics* comes to us increased to eighty pages, and is especially interesting and attractive. It contains, besides the regular monthly contributions by Jacobi on the "Therapeutics of Infancy and Childhood," and Forchheimer on the "Medical Diseases of the Mouth," either one of which is worth the year's subscription, Townsend on "Acute Lobar Pneumonia in Children," Seibert on "Stomach Washing of Infants," an interesting article (illustrated) on the latest procedure in the treatment of Gastro-intestinal Catarrh, Baruch on the "Treatment of Incontinence of Urine," Earle on "Diphtheria in Chicago," Keating on the "Differential Diagnosis in the Fevers of Childhood," and a large number of brief, practical abstracts from the German, French, and English medical journals of the day.

---

NOW READY—Transactions of the American Association of Obstetricians and Gynecologists. Vol. I.: 1888. Illustrated. Price, $5.00; Half Russia, $6.00. May be obtained of the publisher, William J. Dornan, 100 North Seventh street, Philadelphia (see adv. page 10), or through booksellers. In Buffalo, of Peter Paul & Bro., 420 Main street, where this journal may also be obtained. Orders may also be addressed to the Secretary, William Warren Potter, M. D., 284 Franklin street, Buffalo, N. Y.

---

THE COSMOPOLITAN is one of the best magazines that can be printed, and should find a place on every library table. Attention is called to the combination offer that is made to new subscribers on adv. page 22. The *Cosmopolitan* and the BUFFALO MEDICAL AND SURGICAL JOURNAL will be furnished to new subscribers for $3.00 a year.

---

CONGRATULATIONS.—Inset advertisements will be discontinued after this issue.—*Canadian Practitioner*, March 1st. Next!

---

PHYSICIANS in search of a location, or contemplating a change thereof, will find something of interest on adv. page 26.

